# Percutaneous cruciate repair of ruptured Achilles tendon

**DOI:** 10.1186/s13018-023-04167-9

**Published:** 2023-09-12

**Authors:** Nicola Maffulli, Giuliano Sammaria, Salvatore Ziello, Filippo Migliorini, Francesco Oliva

**Affiliations:** 1 Department of Musculoskeletal Disorders, Faculty of Medicine and Surgery, University of Salerno, 84084 Baronissi, Italy; 2 Clinica Ortopedica, Ospedale San Giovanni di Dio e Ruggi d’Aragona, 84131 Salerno, Italy; 3 Centre for Sports and Exercise Medicine, Barts and the London School of Medicine and Dentistry, Mile End Hospital, Queen Mary University of London, 275 Bancroft Road, London, E1 4DG UK; 4 School of Pharmacy and Bioengineering, Faculty of Medicine, Keele University, Thornburrow Drive, Stoke on Trent, ST4 7QB UK; 5 Department of Orthopaedic, Trauma, and Reconstructive Surgery, RWTH University Medical Centre, Pauwelsstraße 30, 52074 Aachen, Germany; 6 Department of Orthopedics and Trauma Surgery, Academic Hospital of Bolzano (SABES-ASDAA), 39100 Bolzano, Italy; 7 Department of Human Sciences and Promotion of the Quality of Life, San Raffaele Open University, Rome, Italy

**Keywords:** Percutaneous, Achilles tendon, Cruciate suture, Tendon repair

## Abstract

Percutaneous repair is a safe and reliable method to restore continuity after acute Achilles tendon ruptures, with a lower incidence of wound complications compared to open techniques. We describe a novel percutaneous cruciate suture performed through 5 stab skin incisions, four of which are longitudinal and parallel to the course of the sural nerve to minimize the risk of injury and one transverse incision at the site of rupture, with a total of 16 suture threads and the knot outside the tendon body, increasing the tensile strength of the suture and minimizing the risk of re-rupture. Clinical studies are necessary to ascertain whether the theoretical advantages of the cruciate suture technique translate into better clinical outcomes compared to established percutaneous techniques.

## Introduction

Acute Achilles tendon ruptures (ATRs) occur more frequently in men than women, with a male-to-female ratio from 1.7:1 to 30:1 [1]. Patients aged 40–59 years mostly suffer (78%) from such injuries [2]. Several risk factors have been identified: gender, age, being overweight, blood group, HLA-types, diabetes, and therapeutic agents (corticosteroids, fluoroquinolone, antibiotics, weight-lowering drugs) [3–7]. Tendon fibres begin to disrupt after a length increase of 3–4% and frankly rupture after an increase of 8% [8]. The tear is usually located within 2–7 cm proximal to the Achilles tendon insertion on the calcaneus [9]. Acute Achilles tendon ruptures can be treated surgically, using open, minimally invasive, endoscopically assisted and percutaneous approaches, or non-operatively [10]. Non-operative management of AT ruptures was first described in 1575 by Ambroise Pare and classically consists of immobilization in an equinus cast. Conservative management has a reported re-rupture rate of up to 17% and may result in significant loss of strength and power in the calf muscles [11–13]. Open surgical management of patients with ruptured Achilles tendons allows accurate apposition of the tendon stumps but can lead to difficulty in wound healing and infection [14]. Minimally invasive approaches to repair the Achilles tendon have emerged as viable alternatives for acute Achilles tendon ruptures, reducing the rate of complications [15–17]. The operative incision (average length of approximately 10 cm) and the handling of the Achilles tendon tissue required for open surgery may affect postoperative recovery [18]. A percutaneous technique was first described by Ma e Griffith in 1997, consisting of a Bunnell suture applied in the proximal tendon and a box suture in the distal stump inserted through six para-tendinous stab incisions, three lateral and three medial to the AT [19]. In 1999, Webb and Bannister described a percutaneous technique with three midline transverse 2.5 cm incisions over the posterior aspect of the Achilles tendon [11]. This technique was then modified by McClelland and Maffulli in 2002, maintaining three midline transverse incisions over the Achilles tendon, passing a Mayo needle transversely through the incision and the substance of the tendon, then out again through the same incision, both distally and proximally, in a Kessler configuration [8]. In 2008, Maffulli and Carmont described a new technique with 4 proximal stab incision, 2 distal longitudinal stab and a transverse incision in the gap [20].

**Fig. 1:**
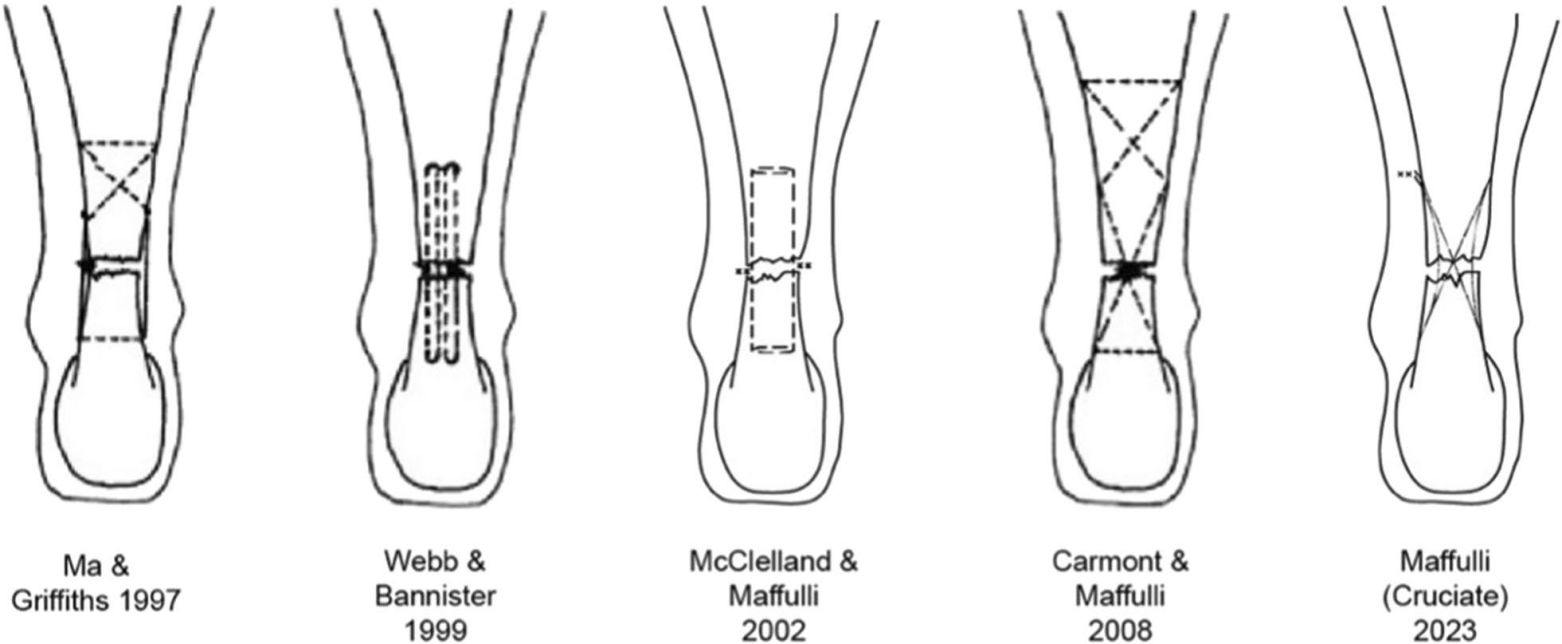
Percutaneous Achilles tendon repair over time

A prospective multicenter randomized controlled study comparing open and percutaneous repair techniques reported a higher rate of re-rupture in patients treated by an open technique (6% vs. 3% using a percutaneous technique). The difference, however, was not statistically significant [21]. Percutaneous repair, compared to open operative repair, is associated with a shorter operation duration and a lower risk of infection [22]. We describe a novel percutaneous suture configuration for Achilles tendon ruptures (Fig. 1).

## Material and methods

**Fig. 2:**
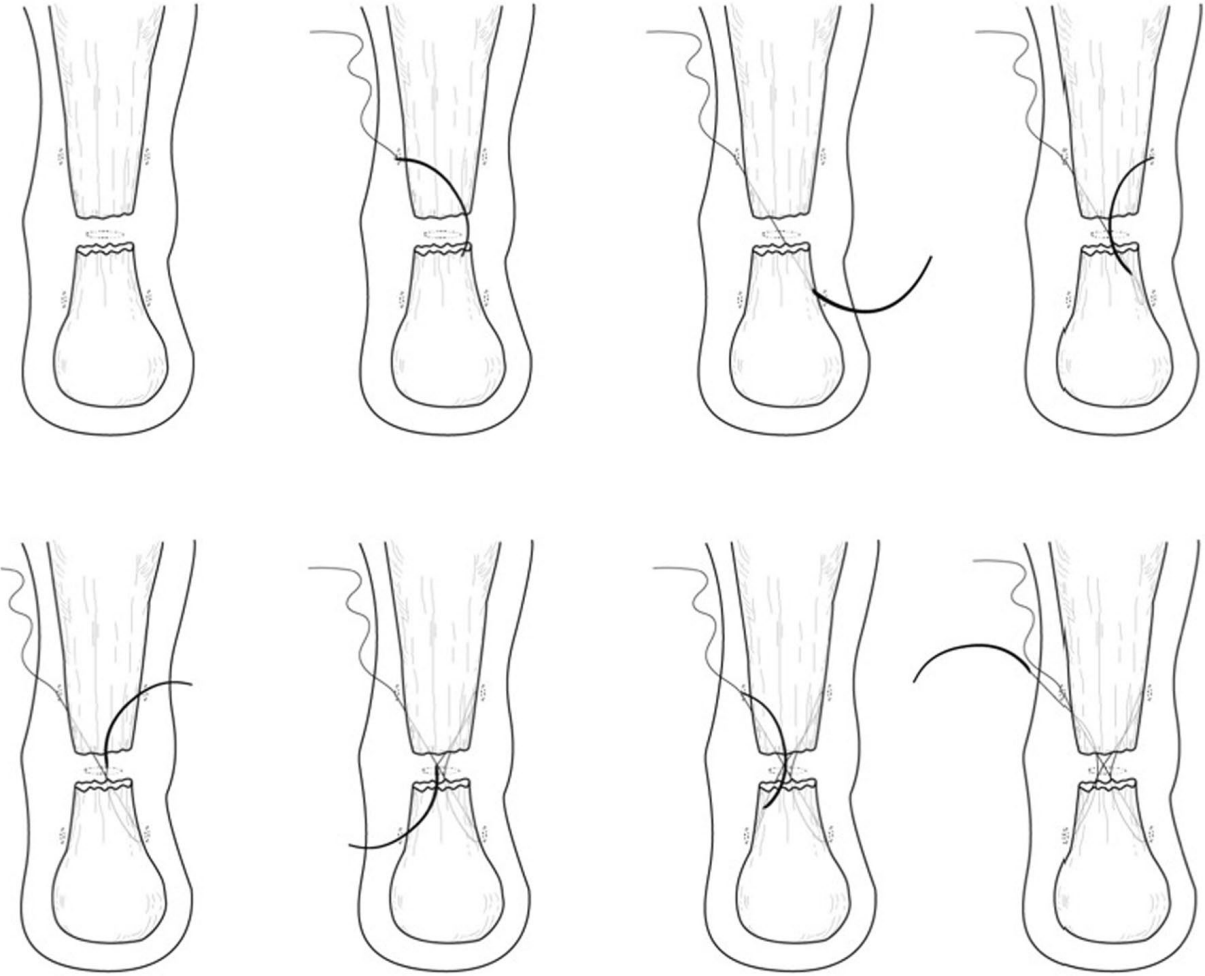
Surgical procedure step by step

The technique consists of six steps (Fig. 2).

*Step 1* Patient positioning.

*Step 2* Skin preparation.

*Step 3* Incision.

*Step 4* Mobilization of the tendon.

*Step 5* Cruciate suture.

*Step 6* Immobilization.

### Surgical procedure

The patient is positioned prone. Skin preparation is performed in the usual fashion and a sterile field is prepped. The areas 4–6 cm proximal and distal to the palpable tendon defect and the skin over the defect are infiltrated with 20 ml of local anaesthetic (10 ml of lidocaine 1% and 10 ml of chirocaine 0.5%). Ten ml of chirocaine 0.5% are infiltrated deep into the palpable tendon defect. A tourniquet is not required.

A 1-cm transverse incision is made over the palpable defect using a size 11 scalpel blade. Two longitudinal stab incisions are made lateral and medial to the tendon 6 cm proximal to the palpable defect. Two further longitudinal incisions on either side of the tendon are made 4–6 cm distal to the palpable defect or just above the insertion of the tendon on the calcaneus. A small, curved mosquito is then used to gently mobilize the tendon from the subcutaneous tissues.

A 9-cm Mayo needle is threaded with 2 double loops of number 2.0 Vicryl (Ethicon, Somerville, New Jersey, USA), and this is passed between the lateral proximal stab incision and the medial distal stab incision, through the transverse incision, through the body of the tendon. Maximum care should be taken, as the body of the tendon is surprisingly superficial. Then, passing again through the transverse incision, we first reach the medial proximal stab incision, and then the lateral distal stab incision. A subsequent diagonal pass is, then, made to the transverse incision over the ruptured tendon to carry the Vicryl laterally and proximally. The suture is then tested for security by pulling with both ends of the Vicryl proximally.

Each of the sutures is then passed distally from just proximal to the transverse Vicryl passage through the bulk of the tendon to pass out of the diagonally opposing stab incision. A clip is used to hold the first throw of the lateral side to maintain the tension of the suture. This suture is then tested for security by pulling with both ends of the Vicryl distally. The ankle is held in full plantar flexion, and the opposing ends of the Vicryl thread are tied together with a double throw knot, and then three further throws before being buried using the forceps.

A subcuticular Monocryl (Ethicon, Somerville, New Jersey, USA) suture is used to close the transverse incision. We do not close the longitudinal stab incisions. Finally, a cast is applied in full plantar flexion.

**Fig. 3:**
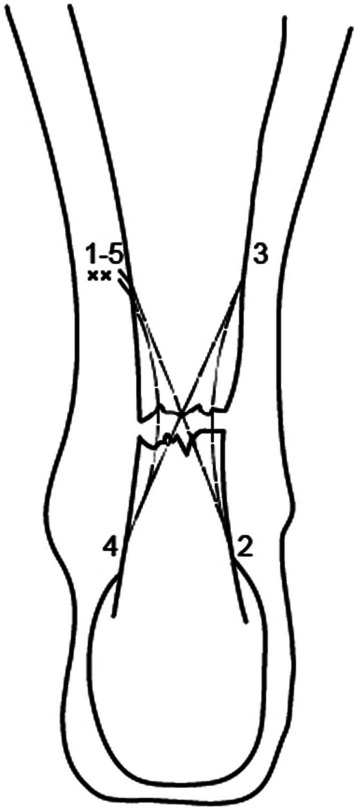
Percutaneous cruciate repair of ruptured Achilles tendon

The patient is discharged on the day of surgery and can fully bear weight in the cast with the foot in full plantar flexion. The cast is removed 2 weeks after the surgery, and the wounds are inspected allowing proprioception, plantar flexion, inversion, and eversion exercises (Fig. 3).

### Post-operative protocol

After surgery, patients are usually discharged the same day, after assessment by a physiotherapist. The full cast is retained for 2 weeks. Then, the cast is removed and an Aircast boot orthosis (XP Walker, DJO Ltd, Guilford, England, United Kingdom) is applied with 5 heel wedges with the foot in maximum plantar flexion. Full weight bearing in the Aircast boot is allowed. After 4 weeks, one wedge is then removed. The boot with the 4 wedges is kept for 4 weeks. From the eighth week after the surgery, and over the course of 2 further weeks, patients are instructed to gradually discard the brace, removing it from 1 to 4 h in the morning and again during the afternoon. At this stage, when not wearing the brace, patients are bearing weight on the operated leg, and are instructed to use a 15 mm heel wedge. At full removal of the brace, patients are required to use the 15 mm heel wedge for another month, at which time they have normally regained a plantigrade ankle. Only at that stage (after approximately 12 weeks), they are allowed to start eccentric exercises of the gastro-soleus complex. Plyometric exercises are permitted at 5 months after the index procedure, and patients are allowed to return to their normal activity, including sports when they feel confident with doing so [23]. Recent studies demonstrate the critical role of functional rehabilitation in the management of ruptured Achilles tendons, and physiotherapy follow-up for gentle mobilization is arranged [24].

## Discussion

The diagnosis of an acute Achilles tendon rupture is based on history and physical examination, without the adjunct of imaging [25, 26]. Patients with an acute rupture of the tendon often describe a popping sound in the posterior aspect of the leg while dorsiflexing the ankle or the sensation of being kicked in the back of the ankle. According to the American Academy of Orthopaedic Surgeons Clinical Practice Guidelines, the diagnosis of an acute Achilles tendon rupture can be established by two or more of the following clinical tests [27]: a positive calf-squeeze test, decreased plantar flexion strength, presence of a palpable defect, and increased passive ankle dorsiflexion with gentle manipulation, or flexion of the knee.

Several clinical tests have been described to diagnose AT rupture. The calf-squeeze test, described first by Simmonds in 1957 and then by Thompson in 1962, is performed with the patient prone on the examination table with ankles clear of the end of the couch [28, 29]. The examiner squeezes the fleshy part of the calf. Squeezing the calf deforms the soleus muscle, causing the overlying AT to bow away from the tibia, producing plantar flexion of the ankle if the tendon is intact. Maffulli reported a sensitivity of 0.96 and specificity of 0.93 for the calf-squeeze test [26]. The knee flexion test, also called the passive ankle dorsiflexion test, described by Matles in 1975, is performed with the patient-prone [30]. Patients are asked to actively flex their knees to 90°. During this movement, if the foot on the affected side falls into neutral or dorsiflexion, an AT rupture can be diagnosed. Maffulli reported a sensitivity of 0.88 and specificity of 0.85 with this test [26]. Other tests are used to formulate a clinical diagnosis of AT rupture: the sphygmomanometer test described by Copeland, and the needle test described by O’Brien [31, 32]. These two latter tests showed a sensitivity of 0.81 and 0.80, respectively [26]. Garras et al. compared clinical examination with magnetic resonance imaging (MRI) for AT rupture, showing a 100% sensitivity when the calf-squeeze, knee flexion test, and palpable defect were indicative of rupture [33]. With such excellent sensitivity and specificity of easily performed diagnostic examinations, clinical examination is the standard for diagnosis. MRI or ultrasonography can be useful as confirmatory tests, but they are not to be considered mandatory, and surely they are not indicated as the only way to formulate a diagnosis [34, 35].

The repair of acute Achilles tendon ruptures performed with this novel percutaneous technique presents 16 suture threads and the knot outside the tendon body, with little risk of sural nerve damage. The strength of the core suture is a major factor for effective tendon repair [36]. The increased repair gap resistance and strength result from the increased number of strands of suture [37]. Tying knots far from the tendon stump favours a greater tensile strength [38]. The nonlocking nature of the technique allows the final tightening of the repair before the knot is tied [39]. Studies performed in hand flexor tendons repair have shown that using the same suture material, the cruciate suture technique is twice as strong to 2 mm gap formation (44 N) compared to the Kessler (22 N), Strickland (23 N), and Savage repairs (21 N). Ultimate tensile strength is also significantly stronger in the cruciate technique (56 N) compared to the Kessler (28 N), Strickland (35 N), or Savage repairs (32 N) [39]. The longitudinal strand orientation and the number of purchase points in a single loop do not affect the strength of the suture. Considering the longitudinal configuration of intratendinous microcirculation and depending on these mechanical issues, parallel strands instead of cruciate ones are preferable [40]. These considerations apply to hand flexor tendon repair, and it is unclear whether they can be transferable to repair torn Achilles tendons.

This procedure consists of only 5 incisions and 16 strands of Vicryl 2.0, each with an average minimum tensile strength of 5.4 kg. The ultimate strength of the repair is based on the number of core suture strands crossing the repair site [41]. The possible complications occurring after a ruptured AT are reported in Table 1

**Table 1:** Possible complications of cruciate percutaneous repair

Early (peri-operative)	Sural nerve damage Haematoma formation
Intermediate (< 6 weeks)	Infection Wound healing complications
Late (6 weeks to 6 months)	Re-rupture of tendon

The sural nerve passes from the posterior aspect of the calf into the foot. At 9.8 cm proximal to the calcaneum, the nerve crosses the lateral border of the Achilles tendon. Distally, the nerve passes laterally, so that it is 18.8 mm lateral to the lateral border of the Achilles tendon at the level of the Achilles tendon insertion into the calcaneum. The recent literature reports a range of 0–27% of sural neuritis after percutaneous procedures [42–44]. A longitudinal stab skin incision, parallel to the course of the nerve, is used in the surgical technique described in the present article. Thus, given the size and orientation of the incisions, nerve injury is unlikely to occur. (The incisions are parallel to the nerve and posterior to it distally.) If a nerve injury does occur, it is more likely to be a longitudinal neurotomy rather than transverse axonotmesis or neurotmesis. This pattern of injury allows a nerve to regenerate along its sheath and minimizes neuroma formation [20]. Pre- or intra-operative high-resolution real-time ultrasonography could reduce the risks associated with percutaneous Achilles tendon repair, as the sural nerve can be easily visualized by high-frequency ultrasonography [35, 45]. The rate of temporary paraesthesia was 2.6%, and no case of complete sural nerve damage was reported with the use of high-frequency ultrasonography [46]. We, however, do not use intra-operative ultrasonography: the procedure is performed under local anesthesia, and we monitor the status of the sural nerve clinically asking patients whether they experienced paraesthesia in the area of distribution of the nerve with each throw. If this happens, the suture is removed, and the relevant step of the procedure is repeated.

## Conclusion

This novel percutaneous cruciate suture technique presents only 5 stab skin incisions, parallel to the course of the sural nerve to minimize the risk of injury, 16 suture threads and the knot outside the body of the tendon, increasing the tensile strength and reducing the risk of re-rupture. The present work only reports this novel surgical technique. Further outcome studies are necessary, and ideally randomized trials should be performed to ascertain whether the theoretical advantages of the cruciate suture technique translate into clinically relevant differences.

## Data Availability

This study does not contain any data material.
